# Real-world study of efgartigimod in AChR antibody-positive generalized myasthenia gravis: thymus status, multi-domain symptom improvement and steroid-sparing effect

**DOI:** 10.3389/fimmu.2026.1837584

**Published:** 2026-05-22

**Authors:** Kariman Aili, Mingjia Lu, Na Zhao, Mayinuer Maimaiti

**Affiliations:** 1 Neuroimmunology Department, Neurology Diagnosis and Treatment Center, People’s Hospital of Xinjiang Uygur Autonomous Region, Urumqi, China; 2 Xinjiang Clinical Research Center for Stroke and Neurological Rare Disease, Urumqi, China

**Keywords:** efgartigimod, generalized myasthenia gravis, MG-ADL score, QMG score, steroid-sparing effect, thymic abnormality

## Abstract

**Background:**

Efgartigimod has been approved for the treatment of gMG. However, real-world evidence regarding its efficacy across various functional domains in patients with differing thymic statuses, as well as its steroid-sparing effect when used in conjunction with conventional immunotherapy, remains limited.

**Methods:**

This single-center, real-world retrospective study included AChR antibody-positive patients with gMG who underwent at least two cycles of efgartigimod. The primary endpoints were the changes in MG-ADL and QMG scores at weeks 4 and 8. Secondary endpoints encompassed the MSE rate, IgG reduction, subgroup analyses stratified by thymic status and treatment regimens, and improvements in ocular, bulbar, limb, and trunk functional domains. Generalized estimating equations were employed for the analysis of longitudinal data.

**Results:**

A total of 62 patients participated in the study, comprising 32 females and 30 males, with a mean age of 55 ± 14 years. Significant improvements in the MG-ADL (mean: 4.55 points) and QMG scores (mean: 7.52 points) were noted at week 4 (all *P* < 0.001), with these benefits sustained at week 8. After two treatment cycles, 77.4% (48/62) of patients experienced CMI. The MSE rate was significantly higher in the abnormal thymus group at week 4 (67.7% vs. 38.7%, *P* = 0.022), whereas no significant difference was observed between the two groups at weeks 8. Ocular symptoms, particularly ptosis, demonstrated the most substantial improvement, and significant enhancements were also recorded in bulbar and limb functions. Efficacy was comparable across the monotherapy, steroid combination, immunosuppressant combination, and triple therapy groups. Concomitant steroid treatment facilitated a maximum reduction in prednisone dosage of 78%. No adverse events were reported during the study.

**Conclusion:**

Efgartigimod rapidly, significantly, and sustainably alleviates symptoms in patients with AChR antibody-positive gMG, with its efficacy remaining unaffected by thymic status or concurrent immunotherapy. Patients with thymic abnormalities exhibit an earlier therapeutic response; however, long-term outcomes are comparable to those of patients with a normal thymus. The extent of improvement varies across functional domains, with the most pronounced benefits observed in ocular symptoms. Efgartigimod demonstrates independent therapeutic effects and facilitates substantial tapering of steroids when administered alongside glucocorticoids, underscoring its significant clinical value.

## 1 Introduction

Generalized myasthenia gravis (gMG) is an antibody-mediated autoimmune disorder that is characterized by impaired neuromuscular transmission. Approximately 80%–85% of patients with gMG test positive for circulating AChR antibodies, primarily of the IgG1 and IgG3 subtypes. These antibodies disrupt neuromuscular transmission by blocking, cross-linking, or inducing the degradation of postsynaptic AChRs (1, 2). The global prevalence is estimated to be 150–250 cases per million population, with an annual incidence of 8–10 cases per million (3). In China, the incidence is approximately 0.68 per 100,000 individuals (4). Clinically, gMG presents as fluctuating weakness affecting the ocular, bulbar, respiratory, and limb muscles. Acute exacerbations may result in a myasthenic crisis, significantly impairing patients’ daily functioning and quality of life (5). Conventional immunosuppressive therapies, such as glucocorticoids, azathioprine, tacrolimus, and mycophenolate mofetil, provide satisfactory symptomatic control for most patients. However, approximately 20% of individuals either do not respond to standard regimens or cannot tolerate these agents, leading to refractory MG (6). In recent years, novel biological agents have transformed the therapeutic landscape for gMG. The neonatal FcRn is crucial for maintaining the half-life of IgG antibodies, as it rescues internalized IgG from lysosomal degradation and facilitates its return to systemic circulation through pH-dependent binding (2). Targeted blockade of FcRn has emerged as an effective strategy to enhance the clearance of pathogenic autoantibodies. Efgartigimod (ARGX-113) is a human IgG1-derived Fc fragment engineered for high affinity to FcRn under both acidic and neutral pH conditions. This agent competitively inhibits FcRn-mediated IgG recycling, thereby leading to a rapid and sustained reduction in total IgG and AChR autoantibody levels (7, 8). As a validated FcRn antagonist, efgartigimod has shown rapid, cyclic, and repeatable improvements in MG-ADL scale (9) and QMG score (10), accompanied by a favorable safety profile in the phase III ADAPT trial and its open-label extension, ADAPT+ (11). Subsequent real-world studies have further corroborated its rapid onset and sustained clinical benefits across multiple treatment cycles (12, 13). *Post-hoc* analyses of the ADAPT trial also indicated that efgartigimod enhances all key MG-ADL subitems, which encompass ocular, bulbar, and limb function, thereby suggesting a broad therapeutic spectrum for gMG (14). Despite these promising advancements, the significant clinical heterogeneity of gMG remains a critical issue, with the thymus playing a central pathogenic role in AChR-positive gMG. Approximately 80% of patients with AChR-positive MG exhibit thymic abnormalities, primarily characterized by follicular hyperplasia and thymoma. The formation of ectopic germinal centers, B-cell activation, and autoantigen presentation within the thymus are closely associated with autoantibody production (15, 16). Although thymectomy enhances long-term outcomes and reduces the reliance on immunosuppressive therapy in selected AChR-positive gMG patients, its efficacy is influenced by various confounding factors (17, 18). In real-world clinical settings, a substantial proportion of patients do not achieve sustained remission following surgery, indicating ongoing peripheral autoimmune activity (19). Consequently, there is an urgent need for novel targeted therapies that can rapidly eliminate pathogenic IgG and suppress peripheral humoral immune responses in AChR-positive gMG. Real-world evidence regarding the combined use of efgartigimod and conventional immunotherapy, including glucocorticoids and immunosuppressants such as tacrolimus, mycophenolate mofetil, and azathioprine, remains limited. It is unclear whether combination regimens influence the magnitude or durability of clinical improvements achieved with efgartigimod, or whether efgartigimod provides a clinically significant steroid-sparing effect in routine practice. Although clinical trials have established the overall efficacy and safety of efgartigimod, systematic real-world data on its effectiveness and long-term durability across distinct gMG subgroups are still lacking. In this retrospective real-world study, we analyzed clinical data from 62 AChR-positive gMG patients undergoing efgartigimod treatment. We systematically evaluated changes in MG-ADL and QMG scores, serum IgG levels, and the rate of MSE achievement. Additionally, we compared therapeutic outcomes between patients with normal and abnormal thymus, as well as between efgartigimod monotherapy and various combination regimens. This study aims to provide real-world evidence for the individualized application, efficacy prediction, and combination treatment strategies of efgartigimod in Chinese gMG patients, thereby optimizing clinical decision-making and patient management.

## 2 Methods

### 2.1 Study population

This study was a single-center, retrospective, observational cohort investigation conducted at the People’s Hospital of Xinjiang Uygur Autonomous Region. The study protocol received approval from the Hospital’s Ethics Committee (Approval No. KY2026020901). Clinical data were extracted from the institutional Myasthenia Gravis registry and electronic medical records. Written informed consent for the utilization of clinical data was obtained from all participants at the time of registry enrollment. Consecutive patients diagnosed with acetylcholine receptor (AChR) antibody-positive generalized myasthenia gravis (gMG) who underwent at least two cycles of efgartigimod treatment between September 2023 and November 2025 were screened for inclusion.

### 2.2 Inclusion criteria

Patients meeting the following criteria were included in the study (1): classified as II–IVa according to the Myasthenia Gravis Foundation of America (MGFA) clinical classification; (2) aged 18 years or older; (3) tested positive for anti-AChR antibodies; (4) had baseline thymic imaging classification data available. They were stratified based on thymic status determined by contrast-enhanced chest computed tomography (CT) into two groups: those with normal thymic morphology and those with thymic abnormalities such as hyperplasia or thymoma. Patients who had undergone thymectomy within the previous two years were assigned to the thymic abnormality group.

### 2.3 Exclusion criteria

(1) Treatment with rituximab or eculizumab within 6 months before screening; (2) administration of intravenous immunoglobulin (IVIg) or plasma exchange (PLEX) within 1 month before screening; (3) presence of active infectious disease, severe hepatic or renal insufficiency, other significant neurological disorders, or concomitant autoimmune diseases; (4) inability to complete required study assessments; and (5) pregnancy at enrollment or a planned pregnancy during the study period.

### 2.4 Data collection

The baseline demographic and clinical characteristics collected included age, sex, disease duration, body weight, MGFA classification, history of thymectomy (noting the median time from surgery to the first efgartigimod infusion), and concomitant medications (pyridostigmine, prednisone, and immunosuppressants such as tacrolimus, mycophenolate mofetil, and azathioprine). Thymic status was assessed using contrast-enhanced chest CT for all patients. In those who had undergone thymectomy, the presence of thymic abnormalities was verified through postoperative histopathological examination.

### 2.5 Treatment protocol and follow-up

All patients received intravenous efgartigimod at a dose of 10 mg/kg, administered over approximately 1 hour once weekly for 4 consecutive weeks, constituting a single treatment cycle. Clinical assessments were conducted at baseline (Week 0) and at Weeks 1, 2, 3, 4, and 8 following the initiation of treatment. An additional exploratory follow-up was conducted at Week 24 (after at least two treatment cycles) to assess the durability of the clinical response and to monitor for possible symptom fluctuation; this assessment was not intended to compare cumulative cycle effects but rather to observe long-term stability. When in-person visits were impractical, assessments were performed remotely through telephone or video consultations. To reduce inter-rater variability, all scales were administered by the same trained neurologist in accordance with a standardized procedure.

### 2.6 Outcome measures

The primary outcome measures were the changes from baseline in the Myasthenia Gravis Activities of Daily Living (MG-ADL) score and the Quantitative Myasthenia Gravis (QMG) score at Weeks 4 and 8. Secondary outcomes included: (1) the proportion of patients achieving Clinically Meaningful Improvement (CMI) and Minimal Symptom Expression (MSE); (2) the reduction in total serum IgG levels; (3) changes in individual MG-ADL and QMG subitems, categorized by functional domains (ocular, bulbar, and limb/axial); (4) a comparison of efficacy between patients with and without thymic abnormalities; (5) a comparison of efficacy across different concomitant treatment regimens (monotherapy, combination with prednisone, combination with immunosuppressants, or triple therapy); (6) the steroid-sparing effect, assessed by changes in prednisone dosage. CMI was defined as a reduction of ≥2 points in the MG-ADL score and/or ≥3 points in the QMG score from baseline. MSE was defined as a total MG-ADL score of 0 or 1.

### 2.7 Statistical analysis

Statistical analyses were conducted using R version 4.5.2 (R Foundation for Statistical Computing, Vienna, Austria). The Shapiro–Wilk test was utilized to evaluate the normality of continuous variables. Normally distributed data are reported as mean ± standard deviation (*SD*),and comparisons between groups were made using the independent samples *t*-test. Non-normally distributed variables are presented as median (interquartile range, IQR) and analyzed using the Mann–Whitney U test. Categorical variables are summarized as frequencies (percentages) and assessed using the chi-square (*χ*²) test or Fisher’s exact test, as appropriate. For longitudinal analyses of repeated-measures data, generalized estimating equations (GEE) were employed. The model included time (as a categorical variable), thymic status (abnormal vs. normal), and their interaction term, utilizing an exchangeable working correlation structure and robust standard errors. If the interaction term was not statistically significant (*P*>0.10), it was excluded, and a main-effects model was implemented. The model was additionally adjusted for potential confounders, including age, sex, disease duration, baseline score, history of thymectomy, and concomitant immunotherapy. A one-way analysis of variance (ANOVA) was employed to evaluate the changes in MG-ADL and QMG scores among various treatment regimen groups. Multiple comparisons were adjusted using the Bonferroni method, with a corrected *P* < 0.05 deemed statistically significant. All statistical tests were conducted as two-tailed, and the significance threshold was established at *P* < 0.05. Due to the low rate of loss to follow-up (<5%), missing data were addressed through complete-case analysis. Effect sizes are presented as mean differences (MD) accompanied by 95%confidence intervals (*CI*s).

## 3 Results

### 3.1 Baseline characteristics

62 patients with generalized myasthenia gravis (gMG) who were treated at our hospital from September 2023 to November 2025 and received at least 2 cycles of efgartigimod for antibody depletion were enrolled in this study. Among them, 32 were female and 30 were male. The mean age was55 ± 14 years, the mean disease duration was 3.6 ± 4.4 years, and the mean body weight was 71.9 ± 14.5 kg. The baseline MG-ADL score was 6.61 ± 3.76, and the baseline QMG score was 11.92 ± 6.31. Patients were divided into a thymic abnormality group (n = 31,including 15 cases of thymoma and 16 cases of thymic hyperplasia) and a normal thymus group (n = 31) according to thymic status. In the thymic abnormality group, 24 patients (77.4%) had undergone thymectomy within the previous 2 years, whereas no patient in the normal thymus group had a history of this surgery. 14 patients were classified as typeIIa generalized myasthenia gravis, and 48 patients as typeIIb generalized myasthenia gravis (**Table 1**).

**Table 1:** Comparison of baseline clinical characteristics between two groups of patients with AChR antibody-positive generalized myasthenia gravis.

Item	Total patients (n=62)	Abnormality thymic group (n=31)	Normal thymus group (n=31)	Statistic	*P*-value
Age(years,Mean ± *SD*)	55.0 ± 14.0	51.8 ± 13.9	58.1 ± 13.9	*t* = 1.77	0.082
Gender(Female/Male,n)	32/30	13/18	19/12	*χ²* = 2.29	0.130
Disease duration(years,Mean ± *SD*)	3.6 ± 4.4	3.3 ± 4.4	3.9 ± 4.4	*Z* = -0.54	0.591
Body weight(kg,Mean ± *SD*)	71.9 ± 14.5	74.6 ± 10.8	69.1 ± 17.1	*t* = -1.52	0.133
Baseline MG-ADL score(Mean ± *SD*)	6.61 ± 3.76	6.32 ± 3.26	6.90 ± 4.23	*Z* = -0.61	0.544
Baseline QMG score(Mean ± *SD*)	11.92 ± 6.31	10.58 ± 5.32	13.26 ± 7.00	*t* = 1.69	0.095
History of thymectomy[n(%)]	24	24 (77.4)	0(0)	*χ²* = 37.1	<0.001

### 3.2 IgG reduction rate and proportion of patients achieving MSE status

**Figure 1:**
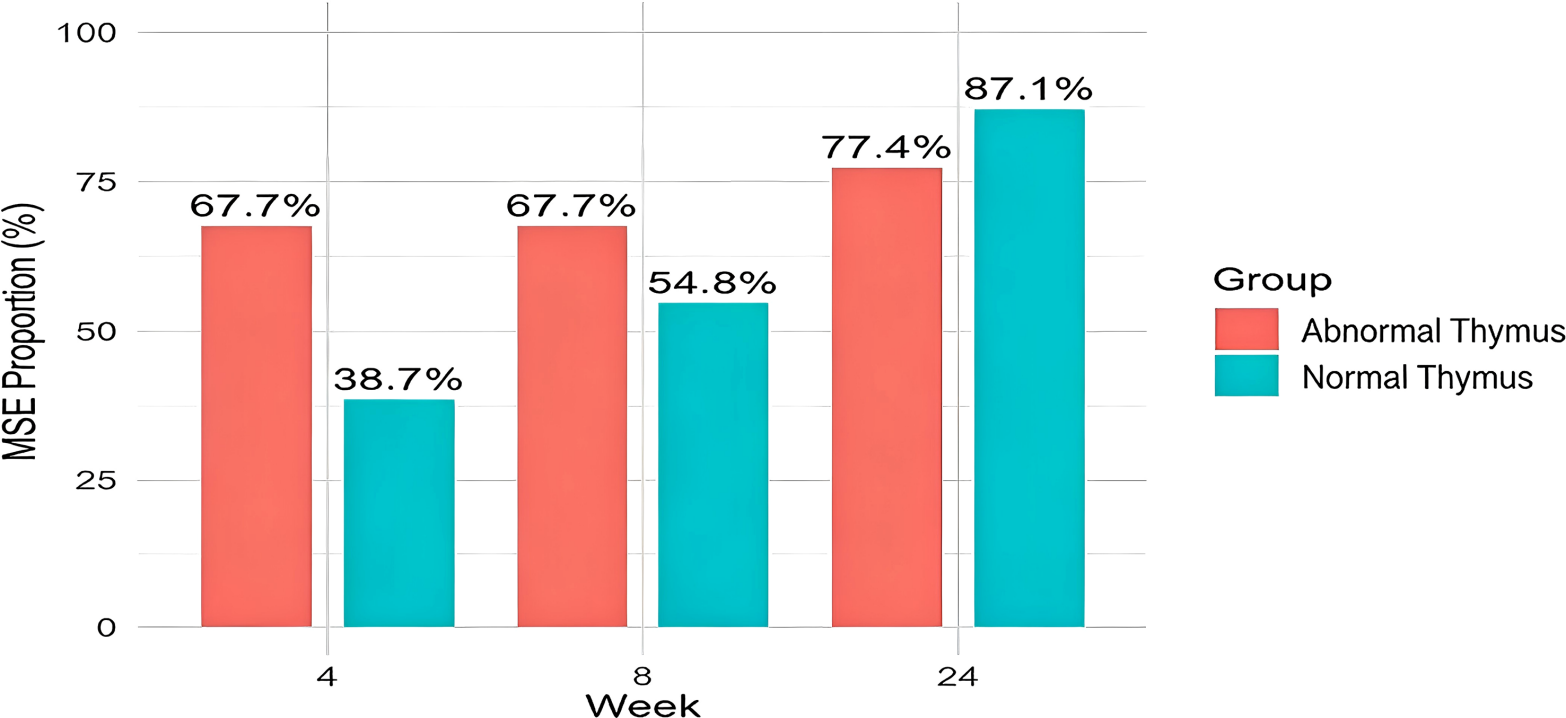
Proportion of patients achieving MSE status in the two groups.

Among the 62 patients, the mean IgG level decreased by 5.34 g/L, corresponding to a 48% reduction from baseline, after one cycle (four infusions) of intravenous efgartigimod. 33 patients (53.2%) achieved MSE status after one cycle of efgartigimod, including 21 patients (67.7%) in the abnormal thymus group and 12 patients (38.7%) in the normal thymus group. The MSE achievement rate was significantly higher in the abnormal thymus group than in the normal thymus group (*p* < 0.05), and this difference was maintained until week 8. After two cycles, 48 patients (77.4%) achieved CMI, including 22 patients (71%), in the abnormal thymus group and 26 patients (83.8%) in the normal thymus group. 38 patients (61.3%) achieved MSE status, including 21 patients (67.7%) in the abnormal thymus group and 17 patients (54.8%) in the normal thymus group. At the 24-week follow-up (after at least two cycles, reflecting longer-term symptom durability), 51 patients (82.3%) achieved MSE status, including 24 patients (77.4%) in the abnormal thymus group and 27 patients (87.1%) in the normal thymus group, with no statistically significant difference between the two groups (*p* > 0.05) (**Figure 1**; **Table 2**).

**Table 2:** Comparison of the proportion of patients achieving minimal symptom expression(MSE)after efgartigimod treatment between the two groups n(%).

Time point	Thymic abnormal	Thymic normal	*χ²*-value	*P*-value
Week 4	21 (67.7)	12 (38.7)	5.20	0.022
Week 8	21 (67.7)	17 (54.8)	1.09	0.297
Week 24	24 (77.4)	27 (87.1)	0.99	0.326

### 3.3 Changes in MG-ADL and QMG scores from baseline in the study population

#### 3.3.1 Changes in MG-ADL and QMG scores

**Figure 2:**
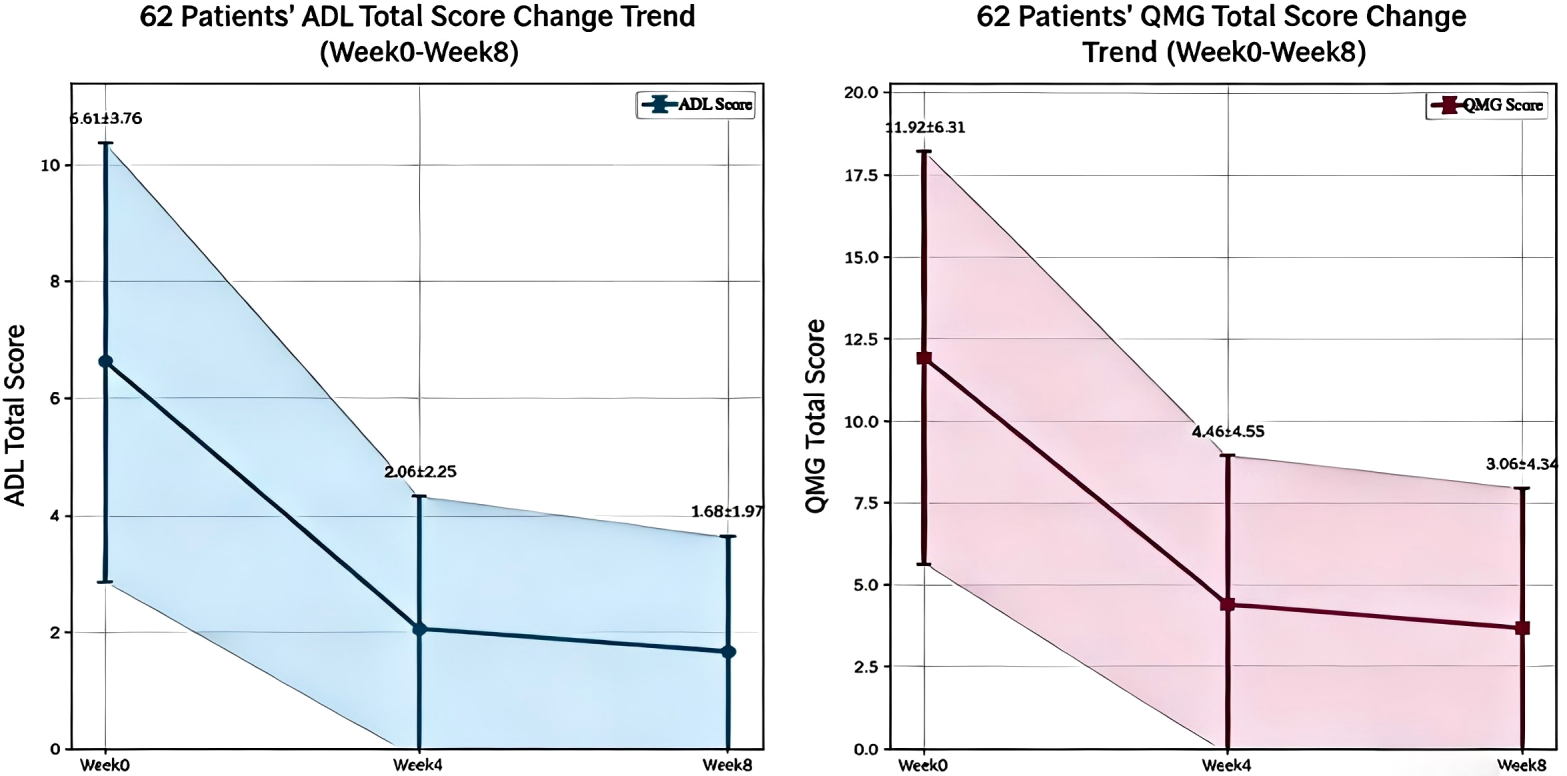
Trends in MG-ADL and QMG scores.

Analysis of total MG-ADL scores in 62 patients was as follows: Baseline (Week0): 6.61 ± 3.76; Week 4: 2.06 ± 2.25, with a mean improvement of 4.55 (95% *CI*: 3.57 to 5.52, *P* < 0.001); Week 8: 1.68 ± 1.97, with a mean improvement of 4.94 (95% *CI*: 3.95 to 5.93, *P* < 0.001). Analysis of total QMG scores was as follows: Baseline (Week 0): 11.92 ± 6.31; Week 4: 4.40 ± 4.55, with a mean improvement of 7.52 (95% *CI*: 5.90 to 9.13, *P* < 0.001); Week 8: 3.66 ± 4.30, with a mean improvement of 8.26 (95% *CI*: 6.55 to 9.96, *P* < 0.001). (**Figure 2**).

#### 3.3.2 Changes in individual MG-ADL subitems from baseline in the study population

**Figure 3:**
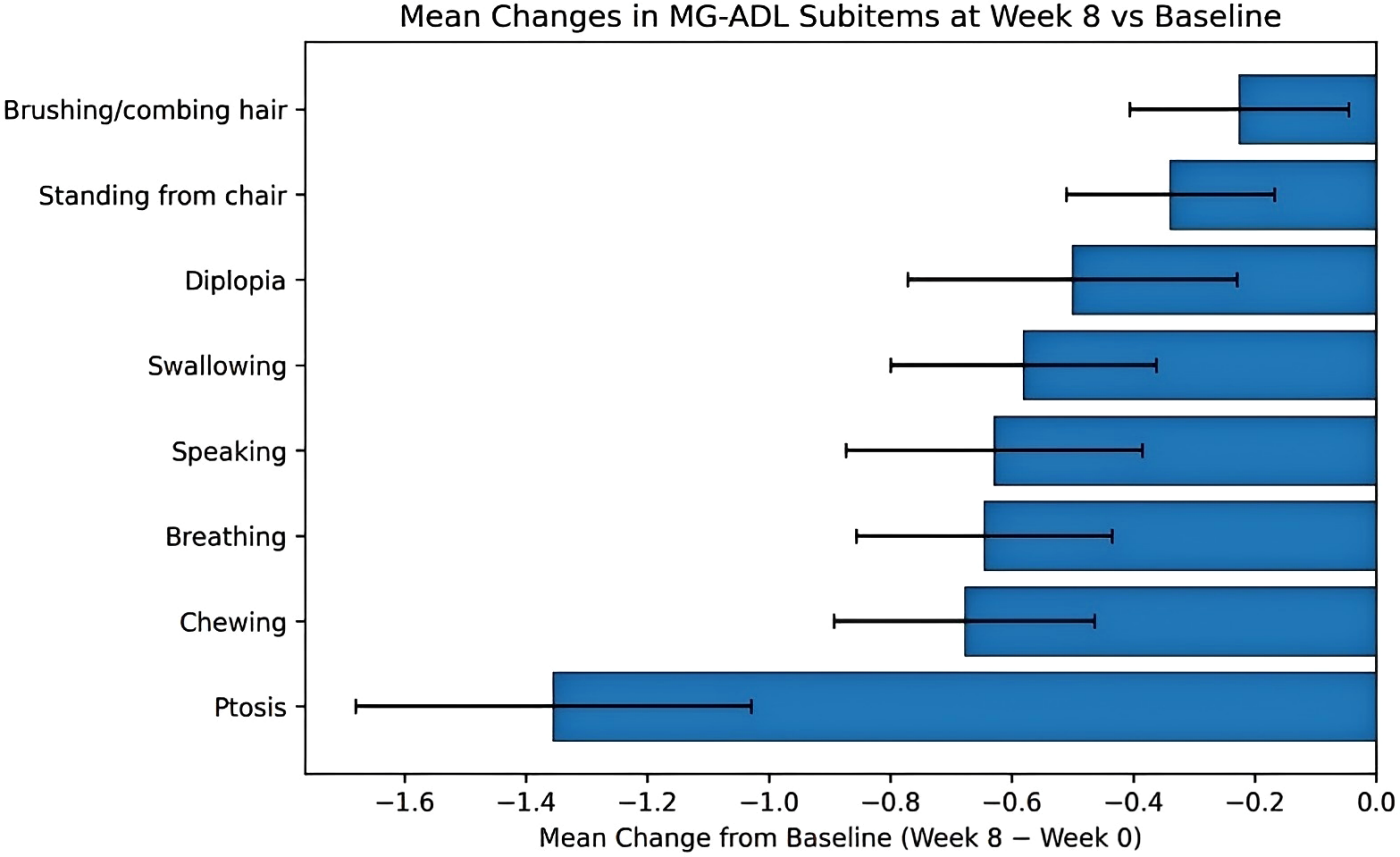
Mean improvements of MG-ADL subitems from baseline to week 8.

Analysis of MG-ADL subitems (Week 0 vs. Week 8) demonstrated that the most significantly improved subitems were as follows: Ptosis (−1.355 ± 1.282, 95% *CI:* −1.680 to −1.029, *t*=−8.32, *P* < 0.001), Chewing (−0.677 ± 0.845, 95% *CI:* −0.892 to −0.463, *t*=−6.31, *P* < 0.001), Breathing (−0.645 ± 0.832, 95% *CI:* −0.856 to −0.434, *t*=−6.11, *P* < 0.001), Speaking (−0.629 ± 0.962, 95% *CI:* −0.873 to −0.385, *t*=−5.15, *P* < 0.001), Swallowing (−0.581 ± 0.860, 95% *CI:* −0.799 to −0.362, *t*=−5.32, *P* < 0.001), Diplopia (−0.500 ± 1.067, 95% *CI:* −0.771 to −0.229, *t*=−3.69, *P* = 0.0005), Standing from a chair (−0.339 ± 0.676, 95% *CI:* −0.510 to −0.167, *t*=−3.94, *P* = 0.0002), and Brushing or combing hair (−0.226 ± 0.711, 95% *CI:* −0.406 to −0.045, *t*=−2.50, *P* = 0.015). The magnitude of improvement was most pronounced for ptosis, and differences in all items were statistically significant (**Figure 3**; **Table 3**).

**Table 3:** Comparison of individual MG-ADL item scores before and after treatment.

Item	Mean	*SD*	95% *CI*	*T*-value	*P*-value
Ptosis	−1.355	1.282	−1.680 to −1.029	−8.32	<0.001
Chewing	−0.677	0.845	−0.892 to −0.463	−6.31	<0.001
Breathing	−0.645	0.832	−0.856 to −0.434	−6.11	<0.001
Speaking	−0.629	0.962	−0.873 to −0.385	−5.15	<0.001
Swallowing	−0.581	0.860	−0.799 to −0.362	−5.32	<0.001
Diplopia	−0.500	1.067	−0.771 to −0.229	−3.69	0.0005
Standing from a chair	−0.339	0.676	−0.510 to −0.167	−3.94	0.0002
Brushing or combing hair	−0.226	0.711	−0.406 to −0.045	−2.50	0.015

#### 3.3.3 Changes in individual QMG subitems from baseline in the study population

**Figure 4:**
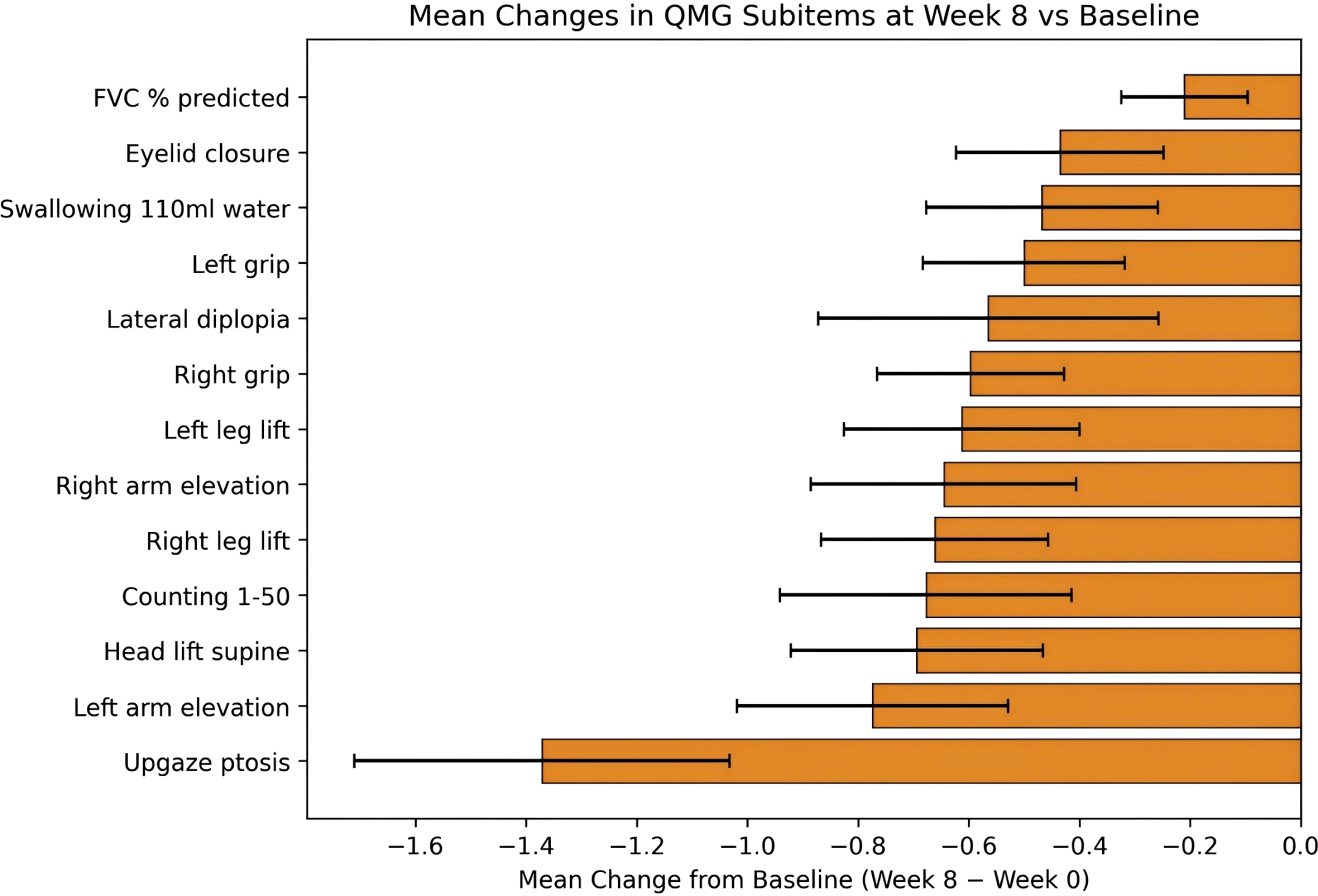
Mean improvements of QMG subitems from baseline to week 8.

Analysis of QMG subitems (Week 0 vs. Week 8) revealed that the most significantly improved subitems were as follows: Ptosis on upgaze (−1.371 ± 1.333, 95% *CI*: −1.710 to −1.032, *t*=−8.10, *P* < 0.001), Duration of left arm elevation to 90° in sitting position (−0.774 ± 0.965, 95% *CI:* −1.019 to −0.529, *t*=−6.32, *P* < 0.001), Duration of head lift to 45° in supine position (−0.694 ± 0.898, 95% *CI:* −0.922 to −0.466, *t*=−6.08, *P* < 0.001), Counting 1–50 (dysarthria) (−0.677 ± 1.037, 95% *CI:* −0.941 to −0.414, *t*=−5.15, *P* < 0.001), Duration of right leg lift to 45° in supine position (−0.661 ± 0.809, 95% *CI:* −0.867 to −0.456, *t*=−6.44, *P* < 0.001), Duration of right arm elevation to 90° in sitting position (−0.645 ± 0.943, 95% *CI:* −0.885 to −0.406, *t*=−5.39, *P* < 0.001), Duration of left leg lift to 45° in supine position (−0.613 ± 0.837, 95% *CI:* −0.825 to −0.400, *t*=−5.77, *P* < 0.001), Right hand grip strength (−0.597 ± 0.664, 95% *CI:* −0.766 to −0.428, *t*=−7.07, *P* < 0.001), Diplopia on lateral gaze at 45° (−0.565 ± 1.210, 95% *CI:* −0.872 to −0.257, *t*=−3.68, *P* = 0.0005), Left hand grip strength (−0.500 ± 0.719, 95% *CI:* −0.683 to −0.318, *t*=−5.48, *P* < 0.001), Swallowing 110 mL water (−0.468 ± 0.824, 95% *CI:* −0.677 to −0.258, *t*=−4.47, *P* < 0.001), Eyelid closure (−0.435 ± 0.738, 95% *CI:* −0.623 to −0.248, *t*=−4.65, *P* < 0.001), and Forced vital capacity as percentage of predicted (−0.210 ± 0.449, 95% *CI:* −0.324 to −0.096, *t*=−3.68, *P* = 0.0005). The improvement was most prominent in upgaze-induced ptosis, and all inter-item differences were statistically significant (**Figure 4**; **Table 4**).

**Table 4:** Comparison of individual QMG item scores before and after treatment.

Item	Mean	*SD*	95% *CI*	*T*-value	*P*-value
Ptosis on upgaze(S)	−1.371	1.333	−1.710 to −1.032	−8.10	<0.001
Duration of left arm elevation to 90°in sitting position(S)	−0.774	0.965	−1.019 to −0.529	−6.32	<0.001
Duration of head lift to 45°in supine position(S)	−0.694	0.898	−0.922 to −0.466	−6.08	<0.001
Counting 1–50(dysarthria)	−0.677	1.037	−0.941 to −0.414	−5.15	<0.001
Duration of right leg lift to 45°in supine position(S)	−0.661	0.809	−0.867 to −0.456	−6.44	<0.001
Duration of right arm elevation to 90°in sitting position(S)	−0.645	0.943	−0.885 to −0.406	−5.39	<0.001
Duration of left leg lift to 45°in supine position(S)	−0.613	0.837	−0.825 to −0.400	−5.77	<0.001
Right hand grip strength(kg)	−0.597	0.664	−0.766 to −0.428	−7.07	<0.001
Diplopia on lateral gaze at 45°(S)	−0.565	1.210	−0.872 to −0.257	−3.68	0.0005
Left hand grip strength(kg)	−0.500	0.719	−0.683 to −0.318	−5.48	<0.001
Swallowing 110 mL water	−0.468	0.824	−0.677 to −0.258	−4.47	<0.001
Eyelid closure	−0.435	0.738	−0.623 to −0.248	−4.65	<0.001
Forced vital capacity as percentage of predicted%	−0.210	0.449	−0.324 to −0.096	−3.68	0.0005

#### 3.3.4 Analysis of improvement characteristics in ocular, bulbar and limb symptoms

Analysis of changes in MG-ADL and QMG subitems at Week 8, compared to baseline, revealed that ocular symptoms—including ptosis, upgaze-induced ptosis, diplopia, lateral gaze-induced diplopia, and eye closure—exhibited the most significant improvement, particularly upgaze-induced ptosis, which showed a reduction of −1.371 ± 1.333 (*P* < 0.001). Bulbar symptoms, encompassing chewing, breathing, speaking, swallowing, dysarthria, swallowing 110 ml of water, and percent predicted vital capacity, also demonstrated significant improvement, with score reductions ranging from approximately 0.2 to 0.68 points (all *P* < 0.001). Indicators of limb function, such as limb lift duration, grip strength, standing from a chair, and brushing/combing hair, exhibited clear and robust enhancements, with upper limb lift and grip strength reflecting relatively greater benefits (maximum reduction −0.774 ± 0.965, *P* < 0.001). A comprehensive comparison indicated that the intervention led to the most substantial improvement in ocular symptoms among patients with myasthenia gravis, followed by bulbar symptoms, while limb function also showed consistent and significant improvements. These findings suggest that the intervention effectively ameliorates the manifestations associated with different muscle groups, with the most pronounced relief observed in ocular muscle-related symptoms (**Table 5**).

**Table 5:** Changes in scale scores of different symptom domains after treatment.

Symptom dimension	Number of subitems	Mean score change(*x^-^ ± s*)	95%*CI*	*P*-value
Ocular symptoms	5	−0.845 ± 1.098	−1.012 to −0.678	*P* < 0.001
Bulbar symptoms	7	−0.555 ± 0.837	−0.689 to −0.421	*P* < 0.001
Limb symptoms	9	−0.561 ± 0.806	−0.675 to −0.447	*P* < 0.001

### 3.4 Comparison of efficacy between abnormal thymus group and normal thymus group

#### 3.4.1 Efficacy in the abnormal thymus group

**Figure 5:**
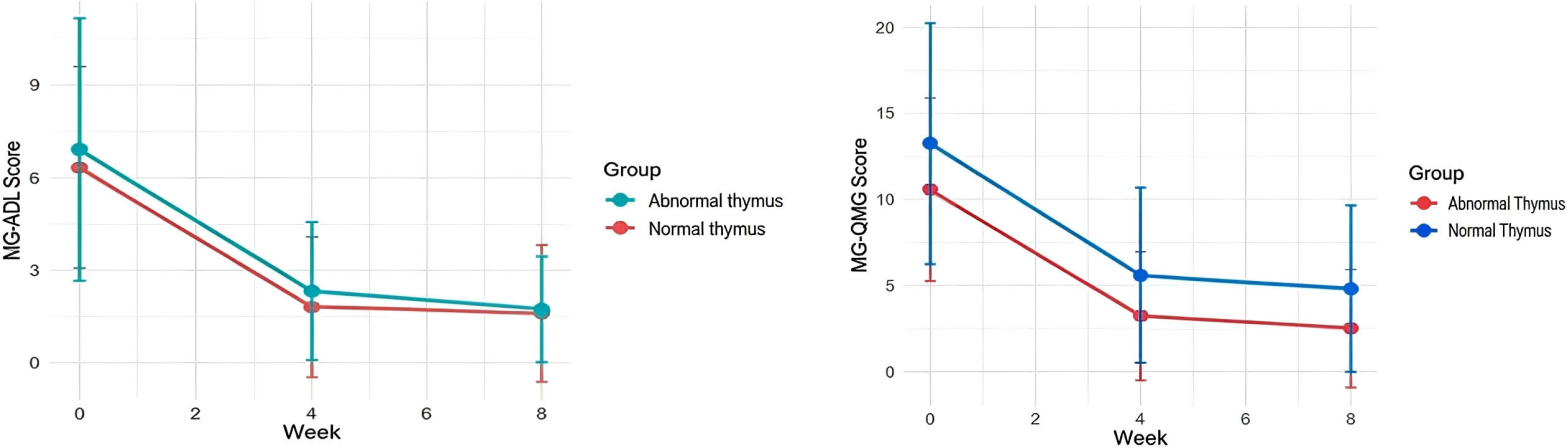
Trends of changes in MG-ADL and QMG scores between the two groups.

A total of 31 patients with generalized myasthenia gravis and abnormal thymus were enrolled in this study, including 13 females and 18 males. The mean age was 51.8 ± 13.9 years, mean disease duration 3.3 ± 4.4 years, and mean body weight 74.6 ± 10.8 kg. Fifteen patients had thymoma and 16 had thymic hyperplasia, among whom 24 patients had undergone thymectomy (Median time from surgery to first infusion: 9.2 months). After one cycle (four infusions) of intravenous efgartigimod, the mean IgG level decreased by 5.3 g/L (47%). In patients with thymoma, the mean IgG level decreased by 5.07 g/L (45%) after one cycle of efgartigimod. In patients with thymic hyperplasia, the mean IgG level decreased by 5.52 g/L (48%) after one cycle of efgartigimod. The baseline MG-ADL score was 6.32 ± 3.26, which decreased significantly at Week 4 (mean difference: −4.52, 95% *CI*: −5.68 to −3.35, *P* < 0.001) and at Week 8 compared with baseline (mean difference: −4.71, 95% *CI*: −5.80 to −3.62, *P* < 0.001). The baseline QMG score was 10.58 ± 5.32, which decreased significantly at Week 4 (mean difference: −7.35, 95% *CI*: −9.08 to −5.63, *P* < 0.001) and at Week 8 compared with baseline (mean difference: −8.06, 95% *CI*: −9.85 to −6.28, *P* < 0.001) (**Figure 5**).

#### 3.4.2 Efficacy in the normal thymus group

The normal thymus group included 31 patients, with a mean age of 58.1 ± 13.9 years, including 19 females and 12 males; mean disease duration was 3.9 ± 4.4 years, and mean body weight was 69.1 ± 17.1 kg. After one cycle (four infusions) of intravenous efgartigimod, the mean IgG level decreased by 5.4 g/L (49%). The baseline MG-ADL score was 6.90 ± 4.23, which decreased significantly at Week 4 (mean difference: −4.58, 95% *CI*: −6.21 to −2.95, *P* < 0.001) and at Week 8 compared with baseline(mean difference: −5.16, 95% *CI*:−6.78 to −3.54, *P* < 0.001). The baseline QMG score was 13.26 ± 7.00, which decreased significantly at Week 4 (mean difference: −7.35, 95% *CI*: −9.08 to −5.63, *P* < 0.001) and at Week 8 compared with baseline (mean difference: −8.45, 95%*CI*: −11.31 to −5.59, *P* < 0.001) (**Figure 5**).

#### 3.4.3 Comparison of efgartigimod efficacy between the two groups

At Week 4 and Week 8 after efgartigimod treatment, MG-ADL and QMG scores in both the abnormal thymus group and normal thymus group were significantly improved compared with baseline. The improvement in MG-ADL score was greater than 2 points, and the improvement in QMG score was greater than 3 points, indicating that efgartigimod was effective in patients of both groups, with no statistically significant difference between the two groups (*P*>0.05) (**Table 6**).

**Table 6:** Longitudinal changes in MG-ADL and QMG scores after efgartigimod treatment between the two groups.

Indicator	Time point	Thymus abnormality group(n=31)	Normal thymus group(n=31)	Statistic	*P*-value
MG-ADL score	Baseline	6.32 ± 3.26	6.90 ± 4.23	*Z=*0.61	0.54
	Week 4	1.81 ± 2.27	2.32 ± 2.23	*Z=*0.89	0.375
	Change(95% *CI*)	-4.52(-5.68 to -3.35)	-4.58(-6.21 to -2.95)	—	Within-group *P* < 0.001
	Week 8	1.61 ± 2.23	1.74 ± 1.71	*Z=*0.26	0.794
	Change(95% *CI*)	-4.71(-5.80 to -3.62)	-5.16(-6.78 to -3.54)	—	Within-group *P* < 0.001
QMG score	Baseline	10.58 ± 5.32	13.26 ± 7.00	*t=*1.69	0.095
	Week 4	3.23 ± 3.69	5.58 ± 5.06	*t=*2.09	0.041
	Change(95% *CI*)	-7.35(-9.08 to -5.63)	-7.68(-9.73 to -5.63)	—	Within-group *P* < 0.001
	Week 8	2.52 to 3.42	4.81 ± 4.82	*t=*2.14	0.037
	Change(95% *CI*)	-8.06(-9.85 to -6.28)	-8.45(-11.31 to -5.59)	—	Within-group *P* < 0.001

### 3.5 Efficacy of efgartigimod in different treatment regimens of myasthenia gravis

#### 3.5.1 Baseline characteristics and treatment regimens

Among the 62 patients, 11 received efgartigimod monotherapy, while 17 were treated with efgartigimod in combination with prednisone (baseline prednisone dose: 25.95 ± 13.5 mg/d; After 2 cycles of treatment, the mean prednisone reduction within 6 months was 18.23 ± 15.88 mg/d). Additionally, 12 patients received efgartigimod alongside immunosuppressive agents, which included 1 patient on azathioprine (100 mg/d), 1 patient on mycophenolate mofetil (1.5 g/d), and 10 patients on tacrolimus (baseline tacrolimus trough concentration: 2.08 ± 0.93 ng/mL). Furthermore, a total of 22 patients were administered efgartigimod in conjunction with both prednisone and immunosuppressive agents, comprising 1 patient on mycophenolate mofetil (1 g/d), 1 patient on azathioprine (25 mg/d), and 20 patients on tacrolimus (baseline tacrolimus trough concentration: 2.20 ± 0.76 ng/mL). The baseline prednisone dose for this group was 31.09 ± 18.76 mg/d, After 2 cycles of treatment, the mean prednisone reduction within 6 months was 24.18 ± 17.80 mg/d.

#### 3.5.2 Changes in IgG levels according to treatment combination

Following one cycle (4 infusions) of intravenous efgartigimod, the efgartigimod monotherapy group exhibited a mean decrease in IgG of 5.34, reflecting a 41% improvement from baseline levels. In the prednisone + efgartigimod group, the mean decrease was 5.43, corresponding to a 50% improvement. The immunosuppressive agent + efgartigimod group demonstrated a mean decrease of 5.51, indicating a 52% improvement, while the prednisone + immunosuppressive agent + efgartigimod group showed a mean decrease of 5.19, representing a 48%improvement.

#### 3.5.3 Changes in MG-ADL scores according to treatment groups

**Figure 6:**
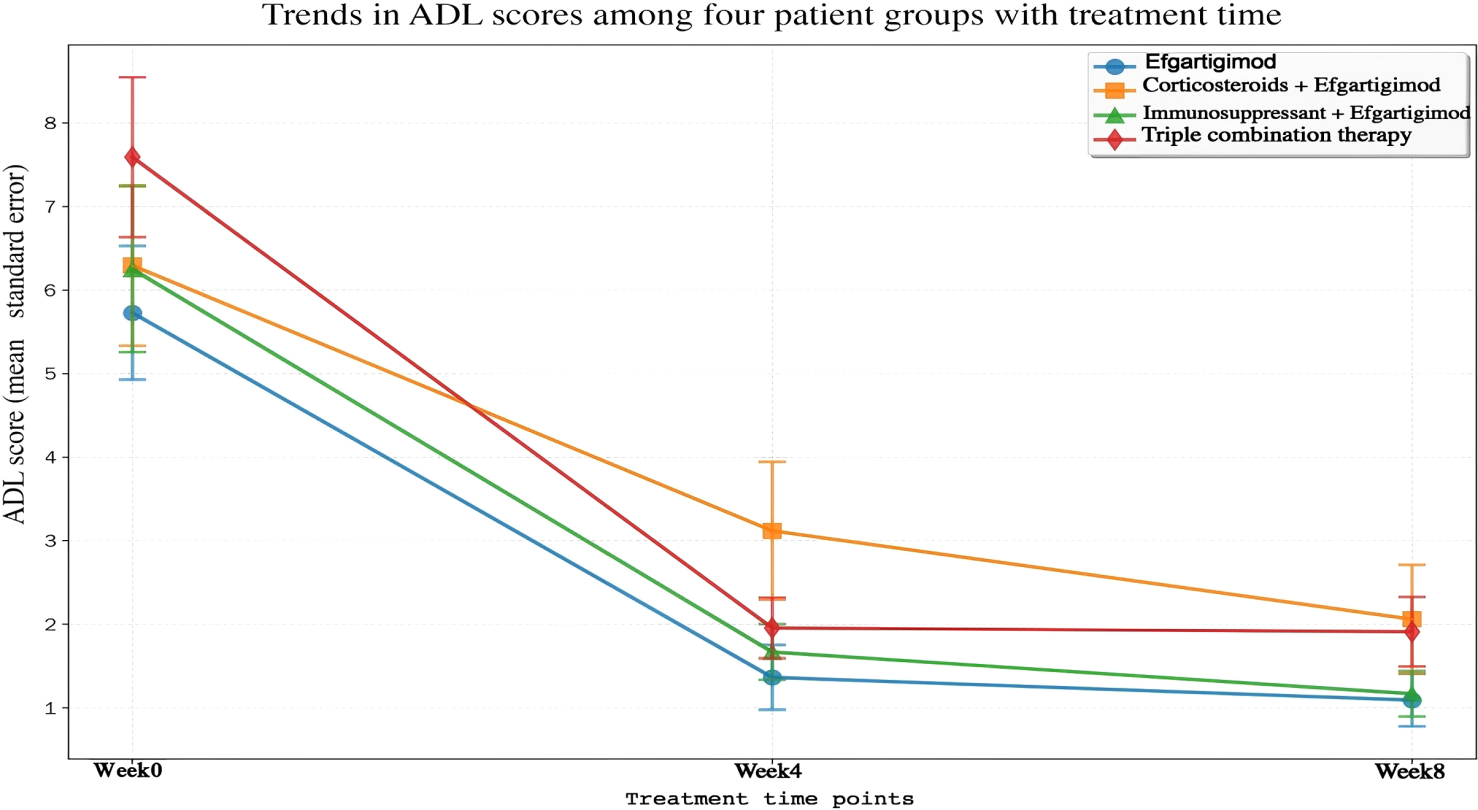
Trends in MG-ADL scores among four patient groups with treatment time.

Efgartigimod monotherapy group (n = 11) had a baseline MG−ADL score of 5.73 ± 2.53. At Week 4, the mean decrease in the MG-ADL score from baseline was 4.36 (95% *CI*: −6.07 to −2.66, *t* = −5.697, *P* = 0.0002); at Week 8, the mean decrease was 4.64 (95% *CI*: −6.26 to −3.01, *t* = −6.355, *P* = 0.0001). The prednisone + efgartigimod group (n = 17) had a baseline MG-ADL score of 6.29 ± 3.85. At Week 4, the mean decrease was 3.18 (95% *CI*: −5.16 to −1.19, *t* = −3.392, *P* = 0.0037); at Week 8, the mean decrease was 4.24 (95% *CI*: −6.43 to −2.04, *t* = −4.094, *P* = 0.0008). In the immunosuppressive agent + efgartigimod group (n = 12), the baseline MG-ADL score was 6.25 ± 3.29. At Week 4, the mean decrease was 4.58 (95% *CI*: −6.87 to −2.29, *t* = −4.405, *P* = 0.0011); at Week 8, the mean decrease was 5.08 (95% *CI*: −7.36 to −2.81, *t* = −4.920, *P* = 0.0005). The triple therapy group (n = 22) had a baseline MG-ADL score of 7.59 ± 4.39. At Week 4, the mean decrease was 5.64 (95% *CI*: −7.68 to −3.59, *t* = −5.730, *P* < 0.0001); at Week 8, the mean decrease was 5.68 (95% *CI*: −7.85 to −3.52, *t* = −5.457, *P* < 0.0001) (**Figure 6**; **Table 7**). Between-group comparison: One-way ANOVA revealed no significant differences in the magnitude of MG-ADL score reduction among the four groups at Week 4 (*F* = 1.265, *P* = 0.295) or Week 8 (*F* = 0.423, *P* = 0.737). These results indicate that there are no statistically significant differences in functional improvement across the various combination regimens.

**Table 7:** MG-ADL score changes following efgartigimod therapy in different treatment groups.

Group	Time point	Score (Mean ± *SD*)	Difference from baseline (95% *CI*)	*T*-value	*P*-value
Efgartigimod	Week0	5.73 ± 2.53	—	—	—
(n=11)	Week4	1.36 ± 1.23	-4.36(-6.07 to -2.66)	-5.697	0.0002
	Week8	1.09 ± 1.00	-4.64(-6.26 to -3.01)	-6.355	0.0001
Prednisone + Efgartigimod	Week0	6.29 ± 3.85	—	—	—
(n=17)	Week4	3.12 ± 3.31	-3.18(-5.16 to -1.19)	-3.392	0.0037
	Week8	2.06 ± 2.60	-4.24(-6.43 to -2.04)	-4.094	0.0008
Immunosuppressant + Efgartigimod	Week0	6.25 ± 3.29	—	—	—
(n=12)	Week4	1.67 ± 1.11	-4.58(-6.87 to -2.29)	-4.405	0.0011
	Week8	1.17 ± 0.90	-5.08(-7.36 to -2.81)	-4.920	0.0005
Triple combination therapy	Week0	7.59 ± 4.39	—	—	—
(n=22)	Week4	1.95 ± 1.66	-5.64(-7.68 to -3.59)	-5.730	<0.0001
	Week8	1.91 ± 1.90	-5.68(-7.85 to -3.52)	-5.457	<0.0001

#### 3.5.4 Changes in QMG scores according to treatment groups

**Figure 7:**
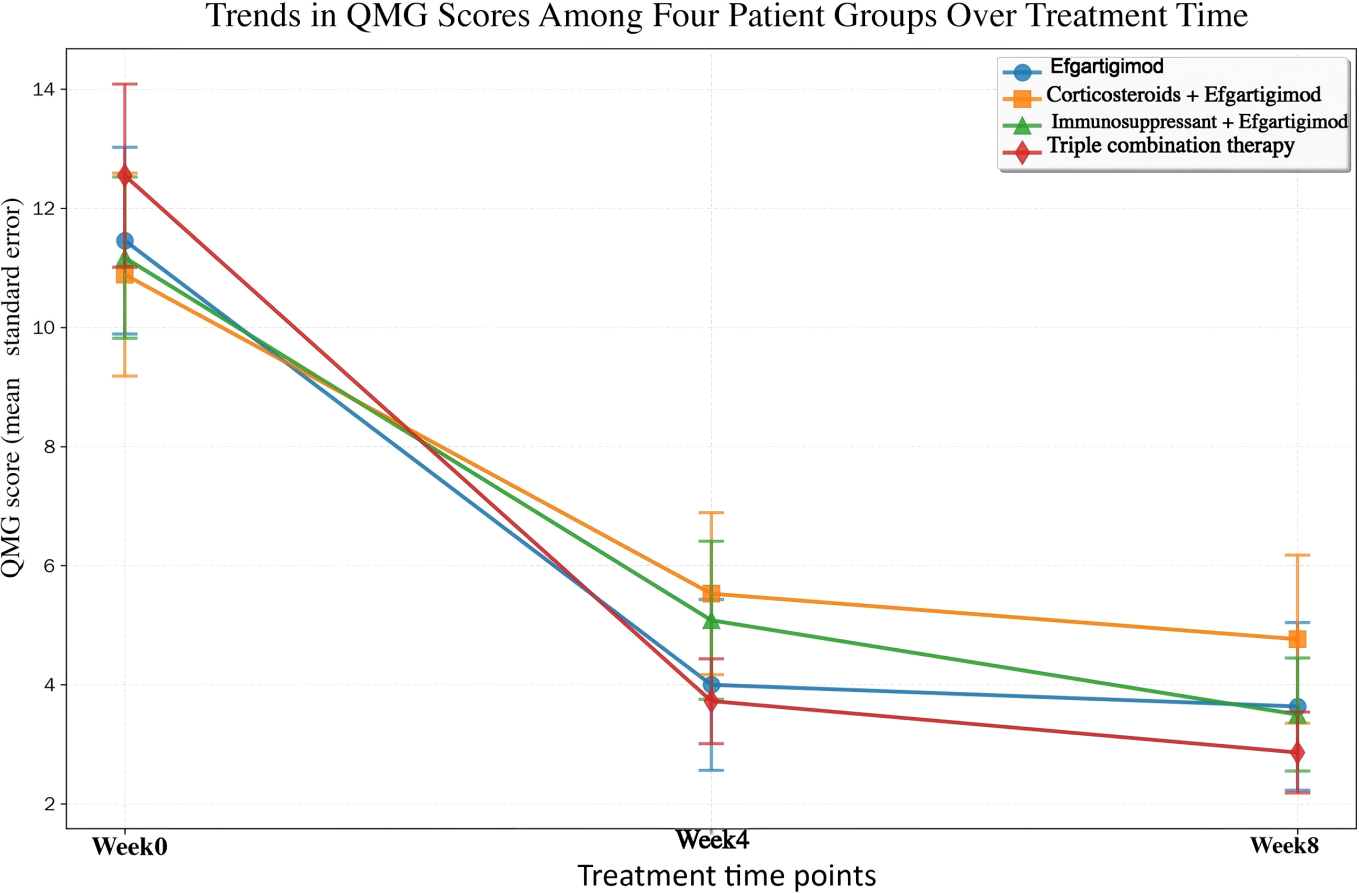
Trends in QMG scores among four patient groups with treatment time.

Efgartigimod monotherapy group (n = 11) exhibited a baseline QMG score of 11.45 ± 4.96. At Week 4, the mean reduction in QMG score from baseline was 7.45 (95% *CI*: −10.45 to −4.46, *t* = −5.546, *P* = 0.0002); at Week 8, the mean reduction was 7.82 (95% *CI*: −11.16 to −4.48, *t* = −5.211, *P* = 0.0004). The prednisone + efgartigimod group (n = 17) had a baseline QMG score of 10.88 ± 5.87. At Week 4, the mean reduction was 5.35 (95% *CI*: −8.24 to −2.47, *t* = −3.933, *P* = 0.0012); at Week 8, the mean reduction was 6.12 (95% *CI*: −9.68 to −2.55, *t* = −3.637, *P* = 0.0022). In the immunosuppressant + efgartigimod group (n = 12), the baseline QMG score was 11.17 ± 4.49. At Week 4, the mean reduction was 6.08 (95% *CI*: −9.01 to −3.16, *t* = −4.580, *P* = 0.0008); at Week 8, the mean reduction was 7.67 (95% *CI*: −10.61 to −4.72, *t* = −5.726, *P* = 0.0001). The triple therapy group (n = 22) had a baseline QMG score of 12.55 ± 7.04. At Week 4, the mean reduction was 8.82 (95% *CI*: −12.26 to −5.38, *t* = −5.333, *P* < 0.0001); at Week 8, the mean reduction was 9.68 (95% *CI*: −13.26 to −6.11, *t* = −5.633, *P* < 0.0001) (**Figure 7**; **Table 8**).Between-group comparison: one-way ANOVA indicated no significant differences between groups regarding the reduction in QMG scores at Week 4 (*F* = 1.149, *P* = 0.337) or Week 8 (*F* = 0.916, *P* = 0.439). These findings further support the notion that various combination regimens demonstrate similar efficacy in enhancing muscle strength.

**Table 8:** QMG score changes following efgartigimod therapy in different treatment groups.

Group	Time point	Score (Mean ± *SD*)	Difference from baseline (95%*CI*)	*T*-value	*P*-value
Efgartigimod	Week0	11.45 ± 4.96	—	—	—
(n=11)	Week4	4.00 ± 4.53	-7.45(-10.45 to -4.46)	-5.546	0.0002
	Week8	3.64 ± 4.46	-7.82(-11.16 to -4.48)	-5.211	0.0004
Prednisone + Efgartigimod	Week0	10.88 ± 5.87	—	—	—
(n=17)	Week4	5.53 ± 5.74	-5.35(-8.24 to -2.47)	-3.933	0.0012
	Week8	4.76 ± 5.64	-6.12(-9.68 to -2.55)	-3.637	0.0022
Immunosuppressant + Efgartigimod	Week0	11.17 ± 4.49	—	—	—
(n=12)	Week4	5.08 ± 4.41	-6.08(-9.01 to -3.16)	-4.580	0.0008
	Week8	3.50 ± 3.15	-7.67(-10.61 to -4.72)	-5.726	0.0001
Triple combination therapy	Week0	12.55 ± 7.04	—	—	—
(n=22)	Week4	3.73 ± 3.26	-8.82(-12.26 to -5.38)	-5.333	<0.0001
	Week8	2.86 ± 3.12	-9.68(-13.26 to -6.11)	-5.633	<0.0001

#### 3.5.5 Comparison of efficacy among different concomitant treatment regimens

Efgartigimod led to significant reductions in IgG levels and enhancements in clinical function, as measured by MG-ADL and QMG scores, regardless of whether patients received concomitant treatment with prednisone or immunosuppressive agents at baseline. In the present study, no statistically significant differences in efficacy were detected among groups, suggesting that Efgartigimod achieves favorable clinical improvements when combined with different background therapies.

### 3.6 Safety evaluation

A total of 62 patients with AChR antibody-positive gMG were enrolled in this study, with all participants receiving a minimum of two cycles of efgartigimod treatment. A standardized protocol for monitoring and collecting adverse events was implemented. During each follow-up, vital signs were routinely assessed, and physical examinations along with laboratory tests—including complete blood counts, liver function, renal function, and other relevant indicators—were conducted. Patients were actively questioned regarding any subjective discomfort, and drug-related adverse reactions were systematically evaluated. Throughout the observation period, no drug-related adverse events were reported among the patients, and no clinically significant abnormalities were detected in vital signs or laboratory parameters. Based on the available clinical data for efgartigimod, common adverse reactions primarily consist of mild to moderate and transient symptoms, such as headache, fatigue, nausea, and upper respiratory tract infections. The incidence of severe adverse reactions, including significant allergic reactions and organ function impairment, remains extremely low. The relatively limited follow-up duration of this study may contribute to an underestimation of adverse event detection. However, in conjunction with the established safety profile and real-world application characteristics of efgartigimod, the absence of adverse events among enrolled patients is clinically justifiable under standardized short-cycle medication and rigorous whole-course clinical management. In conclusion, efgartigimod demonstrates favorable short-term tolerability and safety in this cohort of AChR antibody-positive gMG patients. Further cohort studies with extended follow-up periods are necessary to confirm its long-term safety profile.

## 4 Discussion

This real-world study enrolled 62 patients with AChR antibody-positive gMG to assess the efficacy and safety of efgartigimod. The results indicated that efgartigimod significantly improved both the MG-ADL and QMG scores, with these benefits remaining unaffected by thymic abnormalities or concurrent immunotherapy. After two treatment cycles, 77.4% (48/62) of patients experienced CMI. At Week 4 of treatment, the MG-ADL score decreased from a baseline of 6.61 ± 3.76 to 2.06 ± 2.25, reflecting a mean improvement of 4.55 points (*P* < 0.001). Similarly, the QMG score declined from 11.92 ± 6.31 to 4.40 ± 4.55, corresponding to a mean improvement of 7.52 points (*P* < 0.001), with therapeutic benefits maintained until Week 8. These findings align closely with the results of the phase III ADAPT trial (11) and several published real-world studies (20). Recent real-world evidence has further enhanced the application data for efgartigimod in patients with complex and refractory myasthenia gravis. Multicenter studies focusing on gMG patients who exhibit resistance to or intolerance of intravenous immunoglobulin (IVIg) have demonstrated that efgartigimod can rapidly and significantly improve MG-ADL and QMG scores, achieving a high response rate and a favorable safety profile. Furthermore, it can sustain long-term clinical benefits while reducing steroid dosage. Even among complex patients with severe conditions and multiple comorbidities, efgartigimod is well-tolerated and serves as an effective alternative and rescue therapy for those who experience treatment failure with IVIg (21). Additionally, numerous domestic and international real-world studies have corroborated the stable and reliable efficacy and safety of efgartigimod in gMG populations with varying disease courses, comorbidities, and multiple lines of prior treatment (22, 23), thereby providing robust external evidence to support the conclusions of the present study.

The influence of thymic abnormalities on the efficacy of efgartigimod in the treatment of gMG was assessed in this study, which confirmed all cases of thymic abnormalities through enhanced computed tomography and postoperative pathology. This included 15 instances of thymoma and 16 cases of thymic hyperplasia, thereby ensuring high diagnostic reliability. The primary clinical outcomes revealed that, following efgartigimod treatment, the rate of achieving MSE at Week 4 was significantly higher in the abnormal thymus group compared to the normal thymus group (*P* < 0.05). However, as treatment continued to Weeks 8 and 24, no statistically significant differences in MSE achievement rates were observed between the two groups, indicating that long-term efficacy appeared to be comparable. This dynamic characteristic of efficacy suggests that baseline thymic abnormalities primarily influence the early onset of efgartigimod’s effects, while having a limited impact on mid- and long-term sustained efficacy. The conclusions align closely with those of international multicenter phase II clinical trials. Existing research indicates that patients with thymic abnormality-associated myasthenia gravis (TAMG) and early-onset myasthenia gravis (EOMG), particularly those with thymic hyperplasia, exhibit a higher early clinical response rate following treatment with FcRn-targeted inhibitors. Although a slight decline in symptomatic improvement may occur during the later stages of treatment, the overall condition remains significantly better than at baseline (24, 25). This external evidence further supports the notion that gMG patients with structural thymic abnormalities can achieve rapid early clinical benefits from efgartigimod treatment. This finding provides mutual confirmation between the underlying mechanism and clinical outcomes, particularly regarding the early MSE advantage observed in the abnormal thymus group at Week 4 of this study. From the perspective of autoimmune pathogenesis, the thymus serves as a central immune organ in myasthenia gravis, with structural abnormalities transforming it into a site of persistently activated autoimmune responses. Thymic hyperplasia facilitates the formation of ectopic germinal centers, which continuously induce the activation of AChR-specific T and B lymphocytes, thereby driving the sustained secretion of pathogenic autoantibodies (26). Thymoma epithelial cells aberrantly express autoantigens such as AChR and muscle-specific kinase (MuSK), which directly activate the autoimmune cascade and further exacerbate humoral immune disorders (27). Compared to patients with a normal thymus, individuals with thymic abnormalities typically exhibit a higher baseline autoimmune burden, greater fluctuations in pathogenic IgG levels, and increased disease activity (28). In this highly activated immune microenvironment, efgartigimod rapidly disrupts the imbalanced antibody metabolic homeostasis and reduces the concentration of circulating pathogenic IgG through the targeted clearance of pathogenic antibodies, thereby achieving prompt symptomatic relief. This mechanism underlies the more pronounced early response observed in patients with thymic abnormalities. Regarding mid- and long-term efficacy, the comparable MSE outcomes observed between the two groups at Week 8 and Week 24 in this study align with the long-term follow-up results of the ADAPT trial. Current evidence suggests that the steady-state therapeutic effect of efgartigimod is not influenced by baseline thymic pathological types. Patients with thymoma, thymic hyperplasia, or a normal thymus exhibit similar rates of symptomatic improvement and MSE achievement at the 24-week follow-up (29). Furthermore, real-world studies have corroborated the maintenance of similar mid- and long-term efficacy across patients with various thymic lesions (30). Collectively, the initial therapeutic advantage associated with thymic abnormalities may be gradually counterbalanced by the steady-state effect of sustained antibody clearance by efgartigimod. Consequently, the long-term efficacy of the drug primarily relies on its target mechanism rather than the baseline thymic anatomical status. The early MSE advantage observed in this study is likely attributable to the immune remodeling effects of thymectomy itself, rather than solely determined by the pathological types of the thymus. Baseline data indicated that 77.4% of patients in the abnormal thymus group underwent thymectomy within 2 years prior to enrollment, with a median interval of 9.2 months from surgery to the initiation of medication. In contrast, no patients in the normal thymus group had a history of thymic surgery. As a well-established intervention for AChR-positive gMG, thymectomy can enhance long-term prognosis and decrease the reliance on immunosuppressants (17). However, some postoperative patients continue to experience residual immune disorders, resulting in short-term symptom fluctuations and an inability to achieve complete stable remission (31). Previous clinical studies have demonstrated that gMG patients with a history of thymectomy show a more favorable response to efgartigimod, with early rapid responders predominantly characterized by prior thymectomy, a brief disease course, and severe baseline symptoms (20). Additionally, for gMG patients scheduled for thymectomy, efgartigimod may serve as an effective perioperative bridging therapy, optimizing preoperative conditions, reducing the waiting period for surgery, and minimizing postoperative ICU stays. This approach presents a safe and effective alternative to traditional preoperative interventions such as glucocorticoids, plasma exchange, and intravenous immunoglobulin (32). Consequently, irrespective of thymic status or prior thymectomy history, efgartigimod can swiftly alleviate clinical symptoms and offer a novel option for perioperative and postoperative immunotherapy.

In the structured analysis of symptomatic improvement, functional domains were classified into three primary systems—ocular, bulbar, and limb/axial muscles—according to the classification proposed by Bril V et al. (33), which is based on individual subitems of the MG-ADL and QMG scales. Within the ocular domain, ptosis demonstrated the most significant improvement, with a score reduction from −1.355 to −1.371 (*P* < 0.001), while diplopia exhibited a comparatively milder response, decreasing from −0.500 to −0.565 (*P* < 0.01). This variation indicates heterogeneous responses among different symptoms within the same functional domain. Ocular manifestations, such as ptosis, are highly sensitive to variations in MG-ADL and QMG ratings; thus, even minor enhancements in muscle strength can be quickly reflected in scale scores. The extraocular neuromuscular junction (NMJ) features a simpler structural arrangement, lower AChR density, and reduced expression of complement regulatory proteins, which may collectively contribute to its rapid response to efgartigimod. The decrease in circulating AChR antibody levels following infusion inhibits membrane attack complex (MAC) formation and promotes the repair of extraocular muscles, thereby alleviating ocular symptoms (34). Consistent with our findings, *post-hoc* analyses of the ADAPT trial (24) and a real-world Italian study (20) demonstrated significant improvements across all functional subdomains. Notably, all subitems within the bulbar domain, including mastication, respiration, speech, and swallowing, exhibited robust and uniform enhancements (score reduction: −0.468 to −0.677, all *P* < 0.001), which are clinically significant for mitigating the risk of aspiration pneumonia and malnutrition. The limb/axial domain, which includes arm elevation, grip strength, and chair rising, also showed substantial benefits, with upper limb lifting reflecting the most pronounced improvement (−0.774 ± 0.965, *P* < 0.001). However, the overall magnitude of improvement in this domain was slightly lower than that observed for ocular and bulbar symptoms. These domain-specific outcomes align with *post-hoc* results from the ADAPT trial (14), further confirming that efgartigimod provides broad benefits across systemic muscle groups and enhancing the clinical interpretability of its multi-targeted therapeutic effects.

Patients were categorized into four groups based on combination regimens: efgartigimod monotherapy (11 cases), glucocorticoid plus efgartigimod (17 cases), immunosuppressant plus efgartigimod (12 cases), and triple combination therapy (22 cases). Significant reductions in MG-ADL and QMG scores from baseline were noted at Weeks 4 and 8 across all four groups, with no statistically significant differences observed among them (*P*>0.05). These results suggest that efgartigimod maintains stable and independent clinical efficacy irrespective of concurrent conventional immunotherapy. Notably, regimens that included prednisone exhibited a significant steroid-sparing effect. The dual-combination group achieved a mean reduction in prednisone of 18.23 ± 11.23 mg/day (approximately 58% reduction), while the triple therapy group demonstrated a maximum reduction of 24.18 ± 17.79 mg/day (approximately 78% reduction). This steroid-sparing characteristic holds considerable clinical significance, as prolonged high-dose glucocorticoid exposure is linked to various severe adverse effects, including hyperglycemia, hypertension, osteoporosis, increased infection risk, Cushing’s syndrome, and psychiatric disorders. Multiple real-world studies (35) have similarly corroborated the steroid-sparing potential of efgartigimod. Consequently, the rapid and substantial tapering of steroids facilitated by efgartigimod may enhance long-term outcomes and quality of life for patients with gMG. Regarding safety, no adverse events were reported in the current cohort, aligning with the findings of the ADAPT trial and previous real-world evidence (11).

Several limitations of the present study should be fully acknowledged when interpreting the results. (1) As a single-center retrospective design, this study may introduce selection bias and information bias. (2) The relatively small sample size results in limited statistical power, which may compromise the detection of subgroup differences and rare events. Large-scale, international multicenter cohort studies are warranted for further validation. (3) The diagnosis of thymic abnormalities mainly relied on imaging examinations such as computed tomography (CT), without histopathological confirmation, which may lead to misclassification or missed diagnosis of minor lesions. (4) The follow-up duration was predominantly short-term; thus, long-term outcomes, including delayed autoimmune events, long-term prognosis and disease recurrence, remain unclear. (5) Notably, thymectomy itself can alter the immune progression of the disease and modulate subsequent treatment responsiveness. Accordingly, the early differences in minimal symptom expression (MSE) before and after intervention may be largely attributed to the direct effects of surgery, such as the clearance and remodeling of the immune cell repertoire, rather than merely the pathological characteristics of the thymus. This critical confounding factor requires cautious interpretation of our findings, and all observed differences cannot be simply ascribed to thymic histopathological status. Future prospective studies with rigorous time window matching, multivariate adjustment or propensity score analysis are needed to distinctly differentiate the independent effects of thymic pathology and thymectomy.

## 5 Conclusion

This real-world study demonstrated that efgartigimod produces rapid, profound, and sustained clinical improvements in patients with AChR antibody-positive generalized myasthenia gravis. Significant reductions in MG-ADL and QMG scores were noted as early as Week 4 and persisted through Week 8. After two treatment cycles, 48 of the 62 patients (77.4%) achieved CMI. In the early stages of treatment, patients with thymic abnormalities exhibited a significantly higher rate of MSE compared to those with a normal thymus (67.7% vs. 38.7%, *P* = 0.022). However, no significant difference was observed between the two groups at the 8-week. These findings suggest that patients with thymic abnormalities may experience earlier symptomatic relief, while demonstrating equivalent long-term efficacy compared to those with a normal thymus. Regarding functional domains, ocular symptoms, particularly ptosis, showed the most notable improvement; bulbar symptoms were comprehensively alleviated; and clear therapeutic benefits were also observed in limb and axial muscle function. No significant differences were observed in the improvements of MG-ADL and QMG scores between efgartigimod monotherapy and combination regimens involving glucocorticoids or immunosuppressants. However, combination therapy with glucocorticoids resulted in a substantial reduction in steroid dosage, achieving a maximum decrease of 78%, which indicates a significant steroid-sparing effect. Furthermore, no adverse events were reported in this study, underscoring favorable treatment tolerability. In conclusion, efgartigimod demonstrates reliable efficacy and satisfactory safety in a diverse cohort of AChR antibody-positive gMG patients. Abnormal thymus status does not influence long-term treatment outcomes but is associated with earlier symptomatic remission. Concurrent conventional immunotherapy does not undermine the independent therapeutic effect of efgartigimod and offers additional clinical benefits by facilitating considerable steroid tapering.

## Data Availability

The original contributions presented in the study are included in the article/supplementary material. Further inquiries can be directed to the corresponding author/s.

## References

[1] Phillips WD Vincent A . Pathogenesis of myasthenia gravis: update on disease types, models, and mechanisms. F1000Res. (2016) 5:F1000 Faculty Rev-1513. doi: 10.12688/f1000research.8206.1. PMID: 27408701 PMC4926737

[2] Suzuki S . Pathogenesis and detection methods of anti-acetylcholine receptor antibodies in myasthenia gravis. Immunol Med. (2025) 48:117–23. doi: 10.1080/25785826.2025.2472449. PMID: 40013423

[3] Heldal AT Owe JF Gilhus NE Romi F Aarli JA Skeie GO . Seropositive myasthenia gravis: a nationwide epidemiologic study. Neurology. (2009) 73:150–151. doi: 10.1212/WNL.0b013e3181ad53c2. PMID: 19597135

[4] Chen J Tian D-C Zhang C Li Z Zhai Y Xiu Y . Incidence, mortality, and economic burden of myasthenia gravis in China: a nationwide population-based study. Lancet Reg Health West Pac. (2020) 5:100063. doi: 10.1016/j.lanwpc.2020.100063. PMID: 34327399 PMC8315547

[5] McCallion J Borsi A Noel W Lee J Karmous W Sattler S . Systematic review of the patient burden of generalized myasthenia gravis in Europe, the Middle East, and Africa. BMC Neurol. (2024) 24:61. doi: 10.1186/s12883-024-03553-y. PMID: 38336636 PMC10858594

[6] Uzawa A Kuwabara S Suzuki S Imai T Murai H Ozawa Y . Roles of cytokines and T cells in the pathogenesis of myasthenia gravis. Clin Exp Immunol. (2021) 203:366–74. doi: 10.1111/cei.13546. PMID: 33184844 PMC7874834

[7] Ulrichts P Guglietta A Dreier T van Bragt T Hanssens V Hofman E . Neonatal Fc receptor antagonist efgartigimod safely and sustainably reduces IgGs in humans. J Clin Invest. (2018) 128:4372–86. doi: 10.1172/JCI97911. PMID: 30040076 PMC6159959

[8] Ward ES Gelinas D Dreesen E Van Santbergen J Andersen JT Silvestri NJ . Clinical significance of serum albumin and implications of FcRn inhibitor treatment in IgG-mediated autoimmune disorders. Front Immunol. (2022) 13:892534. doi: 10.3389/fimmu.2022.892534. PMID: 35757719 PMC9231186

[9] Muppidi S . The myasthenia gravis‐Specific activities of daily living profile. Ann N Y Acad Sci. (2012) 1274:114–9. doi: 10.1111/j.1749-6632.2012.06817.x. PMID: 23252905

[10] Bedlack RS Simel DL Bosworth H Samsa G Tucker-Lipscomb B Sanders DB . Quantitative myasthenia gravis score: assessment of responsiveness and longitudinal validity. Neurology. (2005) 64:1968–70. doi: 10.1212/01.WNL.0000163988.28892.79. PMID: 15955957

[11] Howard JF Bril V Vu T Karam C Peric S Margania T . Safety, efficacy, and tolerability of efgartigimod in patients with generalized myasthenia gravis (ADAPT): a multicenter, randomized, placebo-controlled, phase 3 trial. Lancet Neurol. (2021) 20:526–36. doi: 10.1016/S1474-4422(21)00159-9. PMID: 34146511

[12] Krall T Byrne S Kelbert J Chrisman C . Real-world outcomes of efgartigimod in adult myasthenia gravis. Cureus. (2025) 18:e102031. doi: 10.7759/cureus.102031. PMID: 41728453 PMC12923638

[13] Ma C Shen J Zhu Y Wang B Zhu R . Efgartigimod in very-late-onset generalized myasthenia gravis: a real-world study on effectiveness and safety. Curr Neuropharmacol. (2026). doi: 10.2174/011570159X415163251205123550. PMID: 41572778 PMC13519559

[14] Bril V Howard JF Karam C De Bleecker JL Murai H Utsugisawa K . Effect of efgartigimod on muscle group subdomains in participants with generalized myasthenia gravis: post hoc analyses of the phase 3 pivotal ADAPT study. Eur J Neurol. (2023) 31:e16098. doi: 10.1111/ene.16098. PMID: 37843174 PMC11235734

[15] Sikorski PM Kaminski HJ Kusner LL . Thymic hyperplasia in myasthenia gravis: a narrative review. Mediastinum. (2025) 9:17. doi: 10.21037/med-25-12. PMID: 40666538 PMC12260958

[16] Yi JS Guptill JT Stathopoulos P Nowak RJ O’Connor KC . B cells in the pathophysiology of myasthenia gravis. Muscle Nerve. (2018) 57:172–84. doi: 10.1002/mus.25973. PMID: 28940642 PMC5767142

[17] Wolfe GI Kaminski HJ Aban IB Minisman G Kuo H-C Marx A . Randomized trial of thymectomy in myasthenia gravis. N Engl J Med. (2016) 375:511–22. doi: 10.1056/NEJMoa1602489. PMID: 27509100 PMC5189669

[18] Narayanaswami P Sanders DB Wolfe G Benatar M Cea G Evoli A . International consensus guidance for management of myasthenia gravis: 2020 update. Neurology. (2021) 96:114–22. doi: 10.1212/WNL.0000000000011124. PMID: 33144515 PMC7884987

[19] Roberts PF Venuta F Rendina E De Giacomo T Coloni GF Follette DM . Thymectomy in the treatment of ocular myasthenia gravis. J Thorac Cardiovasc Surg. (2001) 122:562–8. doi: 10.1067/mtc.2001.116191. PMID: 11547310

[20] Sgarzi M Paone P Camera G Agazzi E Mazzoleni S Martorana F . Real-world study in Italian public hospital with efgartigimod in patients affected by generalized myasthenia gravis: influence of clinical and serological factors. Front Neurol. (2025) 16:1555068. doi: 10.3389/fneur.2025.1555068. PMID: 40401017 PMC12092212

[21] D'Amico F Campo S Rini N Vinciguerra C Bevilacqua L Erra C . Efgartigimod in patients with generalized myasthenia gravis refractory or intolerant to IVIg. Neurol Ther. (2026) 15(3):1293–1302. doi: 10.1007/s40120-026-00923-1. PMID: 41922674 PMC13172185

[22] Dionísio JM Ambrose P Burke G Farrugia ME Garcia-Reitboeck P Hewamadduma C . Efgartigimod efficacy and safety in refractory myasthenia gravis: UK’s first real-world experience. J Neurol Neurosurg Psychiatry. (2025) 96:322–8. doi: 10.1136/jnnp-2024-334086. PMID: 39798959

[23] Hong Y Yuan CW Lu J Kong PP Huang SQ Yang RY . Efgartigimod for generalized myasthenia gravis in the extreme elderly (≥80 years): a multicenter retrospective real-world study. Front Immunol. (2025) 16:1685233. doi: 10.3389/fimmu.2025.1685233. PMID: 41280898 PMC12631306

[24] Wang S Zhu M Dong J Zhang Y Luo S Jiang J . Perioperative safety and efficacy of efgartigimod for thymoma-associated myasthenia gravis: a prospective, multicenter, phase II clinical trial. J Thorac Oncol. (2025) 20:1120–30. doi: 10.1016/j.jtho.2025.04.014. PMID: 40320172

[25] Jin L Zou Z Wang Q Zeng W Jiang Q Chen J . Patterns and predictors of therapeutic response to efgartigimod in acetylcholine receptor-antibody generalized myasthenia gravis subtypes. Ther Adv Neurol Disord. (2025) 18:17562864251319656. doi: 10.1177/17562864251319656. PMID: 39974170 PMC11837134

[26] Le Panse R Cizeron-Clairac G Cuvelier M Truffault F Bismuth J Nancy P . Regulatory and pathogenic mechanisms in human autoimmune myasthenia gravis. Ann N Y Acad Sci. (2008) 1132:135–42. doi: 10.1196/annals.1405.019. PMID: 18567863

[27] Zhang X Liu S Chang T Xu J Zhang C Tian F . Intrathymic Tfh/B cells interaction leads to ectopic GCs formation and anti-AChR antibody production: central role in triggering MG occurrence. Mol Neurobiol. (2016) 53:120–31. doi: 10.1007/s12035-014-8985-1. PMID: 25407929

[28] Okuno T Koizumi N Yasumizu Y . Pathogenesis of thymoma-associated myasthenia gravis: a narrative review. Mediastinum. (2025) 9:26. doi: 10.21037/med-25-28. PMID: 41098393 PMC12518592

[29] Howard JF Bril V Vu T Karam C Peric S De Bleecker JL . Long-term safety, tolerability, and efficacy of efgartigimod (ADAPT+): interim results from a phase 3 open-label extension study in participants with generalized myasthenia gravis. Front Neurol. (2024) 14:1284444. doi: 10.3389/fneur.2023.1284444. PMID: 38318236 PMC10842202

[30] Hao S Ruan Z Guo R Wang Q Huang X Sun C . Efficacy and safety of efgartigimod for patients with myasthenia gravis in a real-world cohort of 77 patients. CNS Neurosci Ther. (2025) 31:e70391. doi: 10.1111/cns.70391. PMID: 40237260 PMC12001068

[31] AL-Bulushi A Al Salmi I Al Rahbi F Farsi AA Hannawi S . The role of thymectomy in myasthenia gravis: a programmatic approach to thymectomy and perioperative management of myasthenia gravis. Asian J Surg. (2021) 44:819–28. doi: 10.1016/j.asjsur.2020.12.013. PMID: 33579606

[32] Ren L Wei L Jiang S Li H Chen A Zhang J . Efgartigimod for patients with thymoma-associated generalized myasthenia gravis during the perioperative period: a four-case report. Front Immunol. (2025) 16:1627584. doi: 10.3389/fimmu.2025.1627584. PMID: 41112289 PMC12528172

[33] Barnett C Bril V Kapral M Kulkarni A Davis AM . Development and validation of the myasthenia gravis impairment index. Neurology. (2016) 87:879–86. doi: 10.1212/WNL.0000000000002971. PMID: 27402891 PMC5035154

[34] Binu A Kumar SS Padma UD Madhu K . Pathophysiological basis in the management of myasthenia gravis: a mini review. Inflammopharmacology. (2022) 30(1):61–71. doi: 10.1007/s10787-021-00905-9. PMID: 35059932

[35] Goyal N Qi C Stone J Ruck T Wolfe GI Smith AG . Reduction in oral glucocorticoid use after efgartigimod initiation in clinical practice among patients with generalized myasthenia gravis. J Neurol Sci. (2025) 477:123652. doi: 10.1016/j.jns.2025.123652. PMID: 40845416

